# Supplementary material for: Effects of Maternal Supplementation with Organic Trace Minerals including Zinc, Manganese, Copper, and Cobalt during the Late and Post-Partum Periods on the Health and Immune Status of Japanese Black Calves
Source: Animals (Basel). 2023 Nov 28;13(23):3679. doi: 10.3390/ani13233679 (PMC10705794; doi:10.3390/ani13233679)
Supplement: Supplementary file 1 [file animals-13-03679-s001.zip › animals-2660872-supplementary.pdf]

Supplementary Materials

Table S1. Nutrient composition

Item	Last Trimester of Pregnancy	Lactation Period	AI Period
	(Stanchion Barn→ Private Stall)	(Private Stall for breastfeeding)	(Stanchion Herd)
Dry Matter Intake (DM %)	115	118	106
Total Digestible Nutrients (TDN %)	124~125	125	116
Crude Protein (CP %)	113~127	113	111
Formulated Feed A	3~5 kg	5 kg	2 kg
Forages A	0 or 2 kg		
Home-Grown Silages A	6 kg	6 kg	6 kg
Home-Grown Silages B	2 kg	2 kg	
Grass			2 kg
Total mixed rations (TMR)			2 kg

Artificial insemination (AI).

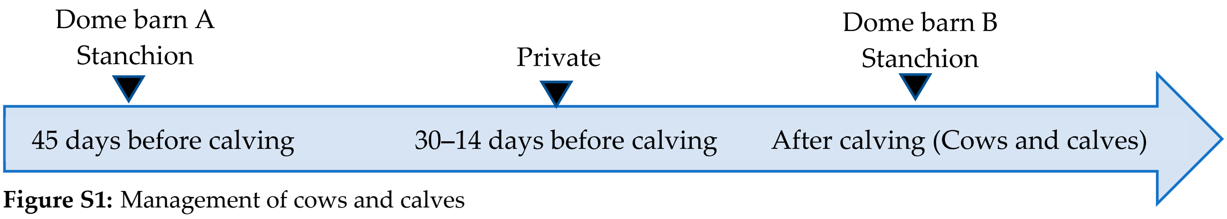

Figure S1: Management of cows and calves

Table S2. The area for each cow and calves **.**

	Dome Barn A left.	Dome Barn B left	Delivery Room
Space	10 m × 21 m = 210 m^2	5 m × 21 m = 105 m^2	3 m × 6 m = 18 m^2
Number of Cows	10–15 cows (accommodate up to 24 animals	7–8 cows (accommodate up to 15 animals)	
Area / Head	14–21 m^2 (10–15 heads)	13.125–15 m^2 (7–8 heads)	18 m^2

The area of dome cowshed A and calving room is calculated for dome barn A left 10 m × 21 m = 210 m^2, holds 10 to 15 cows (Can accommodate up to 24 animals). Area per head: 14–21 m^2 (10–15 heads). Dome barn B left: 5 m x 21 m = 105 m^2, holds 7 to 8 cows (Can accommodate up to 15 animals). Area per head: 13.125–15 m^2 (7–8 heads). In addition, Delivery room: 3 m × 6m = 18 m^2 and area per head: 18 m^2.
